# Comparative Evaluation of the Sealing Ability of Mineral Trioxide Aggregate (MTA)-Based, Resin-Based, and Zinc Oxide Eugenol Root Canal Sealers: An In Vitro Study

**DOI:** 10.7759/cureus.52201

**Published:** 2024-01-13

**Authors:** Anuja Hakke Patil, Amey G Patil, Sabina Shaikh, Sailee Bhandarkar, Anuja Moharir, Anupam Sharma

**Affiliations:** 1 Department of Conservative Dentistry and Endodontics, DY Patil Dental School, Pune, IND; 2 Department of Restorative Dentistry, Rutgers School of Dental Medicine, Newark, USA; 3 Department of Endodontics, Mahatma Gandhi Vidyamandir’s Karmaveer Bhausaheb Hiray Dental College and Hospital, Nashik, IND; 4 Department of Periodontology, DY Patil Dental School, Pune, IND; 5 Department of Conservative Dentistry and Endodontics, Bharati Vidyapeeth Dental College and Hospital, Pune, IND

**Keywords:** zinc oxide eugenol, ah plus sealer, mta fillapex, apical leakage, root canal sealers

## Abstract

Root canal therapy is a crucial procedure in endodontics that is done to achieve complete obliteration of the root canal space. The success of this therapy depends on achieving a proper seal, which is facilitated using root canal sealers. This study aimed to compare the apical sealing ability of three different root canal sealers: MTA Fillapex, AH Plus, and zinc oxide eugenol (ZOE), using the dye penetration method. Forty freshly extracted single-rooted human maxillary incisors were collected and prepared for the study. The root canals were instrumented using the ProTaper system, and the canals were then obturated using the lateral condensation technique with the respective sealers. After one week of storage, the samples were coated with nail varnish, immersed in a rhodamine B dye solution, and then sectioned longitudinally. The depth of dye penetration was measured, and the results were analyzed statistically. The results revealed significant differences in apical leakage among the three experimental groups. Group 2 (AH Plus) showed the minimum leakage with a mean of 0.13 mm, while Group 4 (no sealer) exhibited the maximum leakage with a mean of 4.49 mm. Group 3 (ZOE) showed an intermediate level of leakage with a mean of 2.37 mm. The statistical analysis confirmed the significant difference in mean leakage among the groups.

The findings of this study indicate that AH Plus exhibited superior apical sealing ability compared to MTA Fillapex and ZOE. AH Plus is a resin-based sealer known for its dimensional stability. On the other hand, MTA Fillapex, a newly introduced sealer containing mineral trioxide aggregate, resin, and silica, showed promising sealing properties but had slightly higher leakage compared to AH Plus. ZOE, a traditional sealer, demonstrated relatively higher leakage than the other sealers. In conclusion, choosing a root canal sealer is crucial in achieving a successful endodontic treatment outcome. AH Plus demonstrated superior apical sealing ability among the three sealers tested. Further research and long-term clinical studies are warranted to validate these findings and assess the impact of sealer choice on treatment outcomes and post-endodontic healing.

## Introduction

Root canal therapy is a critical endodontics procedure involving three essential steps: cleaning and shaping, disinfection, and obturation. The ultimate objective of this therapy, as described by Schilder, is the complete obliteration of the root canal space [1]. Failure to achieve a proper seal can lead to the percolation of peri-radicular exudates into incompletely filled canals, resulting in endodontic failure [2].

Gutta-percha is widely regarded as an excellent filling material that fulfills most of the requirements set by Grossman for an ideal obturating material [3]. However, its lack of adhesive qualities to root canal dentin compromises its long-term sealing ability. Root canal sealers facilitate adhesion and close the interface between gutta-percha and root canal dentin. They not only enhance the attainment of a waterproof seal but also serve as a filler for irregularities and discrepancies between the root canal wall and core filling material [4]. Additionally, sealers can traverse lateral and accessory canals, aiding in microbial control when microorganisms remain on the root canal walls or dentinal tubules while acting as lubricants to facilitate thorough seating of the core filling material during compaction [5].

In addition to traditional zinc oxide eugenol (ZOE) sealers, epoxy-based and methacrylate-based resin sealers can be bonded to root canal dentin. AH Plus is an epoxy resin-based sealer commonly used with gutta-percha, known for its adequate long-term dimensional stability [6]. Mineral trioxide aggregate (MTA) has undergone extensive testing and demonstrated many ideal properties. MTA is biocompatible and can be extruded without harmful effects, and it is the only restorative material consistently associated with cementum regeneration [7].

In 2010, an MTA-based sealer, MTA Fillapex, was introduced. It contains MTA, resin, and silica as its main components. It exhibits excellent radiopacity, easy handling characteristics (with a double syringe and self-mixing tips), sufficient working time, and a favorable setting time [8]. Evaluating and comparing the apical sealing ability of this newly introduced sealer with commonly used root canal sealers, such as AH Plus and ZOE, is necessary [9].

Leakage of root canal sealers predominantly occurs between the root canal walls and the sealer. Numerous studies have employed various techniques to quantitatively assess the leakage potential of root canal sealers, including the dye penetration method, radioactive isotope penetration method, electrochemical leakage tests, bacterial penetration, scanning electron microscopy (SEM) analysis, and fluid filtration method [10]. Among these, the dye penetration method is the most commonly used for testing apical leakage. In vitro measurements of dye penetration can be conducted in a linear or volumetric manner [11,12].

This study used the dye penetration method to compare the apical sealing ability of three endodontic sealers, namely, MTA Fillapex, AH Plus, and zinc oxide eugenol. The aim was to identify and recommend a sealer that provides superior apical sealing, creating a favorable environment for post-endodontic healing and ensuring successful treatment outcomes.

## Materials and methods

This in vitro study aimed to compare the sealing ability of three different root canal sealers. The study was conducted at the Department of Conservative Dentistry and Endodontics, Bharati Vidyapeeth Dental College and Hospital, Pune, India.

Sample collection and preparation

The information about the armamentarium utilized in the study, along with the purpose of use and manufacturers, was recorded (Table 1).

**Table 1:** Study materials and instruments used in the study EDTA, ethylenediaminetetraacetic acid; MTA, mineral trioxide aggregate; ZOE, zinc oxide eugenol

Materials and instruments	Purpose of use	Manufacturer
Normal saline	Sample storage	Baxter
Airotor handpiece	Access cavity preparation	Kavo
Endo-access bur		Dentsply Maillefer
K-files	Biomechanical preparation	Mani Inc.
ProTaper system		Dentsply Maillefer
Endo-motor		Dentamerica
5% NaOCl	Irrigation	Hyposept, Sterila
17% EDTA solution	Irrigation	Prime Dental
RC Help	Chelating agent	Prime Dental
Distilled water	Final irrigation	
Paper points	Drying the canal	Dentsply Maillefer
EZ bidirectional filler	Sealer application	Essential Dental Systems
Finger spreaders	Lateral condensation	Mani Inc.
Gutta-percha cones (ISO)	Obturation	Dentsply Maillefer
ZOE	Sealers for obturation	Deepti
AH Plus		Dentsply Maillefer
MTA Fillapex		Angelus
Cavit G	Coronal seal	3M ESPE
Nail varnish	To coat the samples	Maybelline
Incubator	Store samples after obturation	-
Rhodamine B dye	Dye leakage study	Otto
Glass beakers	Dye immersion	Borosil
Diamond disc, micromotor handpiece	Decoronation, sectioning of samples	NSK Ltd.
Stereomicroscope	Dye leakage analysis	Vardhan, Chroma Systems
Digital caliper	To measure root length	Baker
Camera	To take photographs	Sony
Miscellaneous: scissors, cotton pliers, Mini-Endo-Bloc, syringes, mixing pad and spatula, X-ray films, gloves, masks, glass slab, plugger, spirit lamp, condenser, glass rods, and thread		

**Figure 1:**
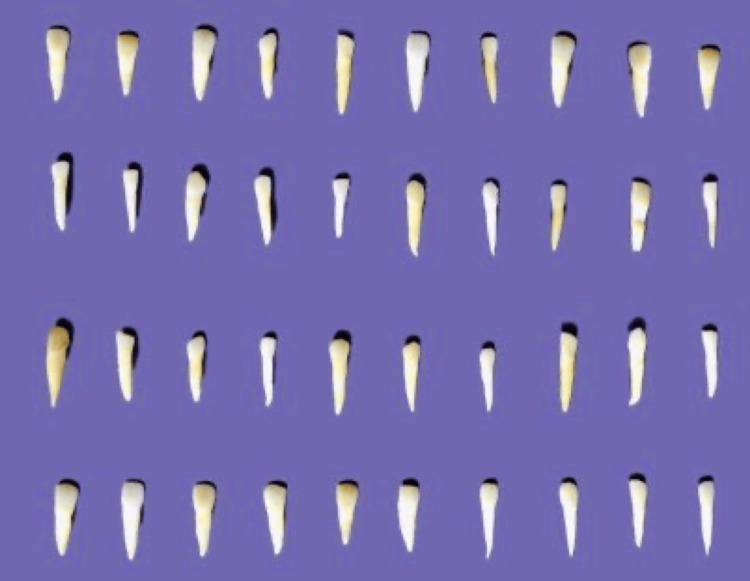
Selected 40 samples before the coronation

Forty freshly extracted single-rooted human maxillary incisors were randomly collected from the Exodontia Department of Bharati Vidyapeeth Dental College, Pune, India. The extracted teeth were immediately washed under running water to remove surface debris, deposits, and calculus. Teeth with external root resorptive defects or cracks, extreme root curvatures, previous root canal filling, caries, root fracture, incompletely formed apex, or hypercementosis were excluded from the study. Only caries-free maxillary incisors with completely formed apices and intact straight roots were selected (Figure 1).

**Figure 2:**
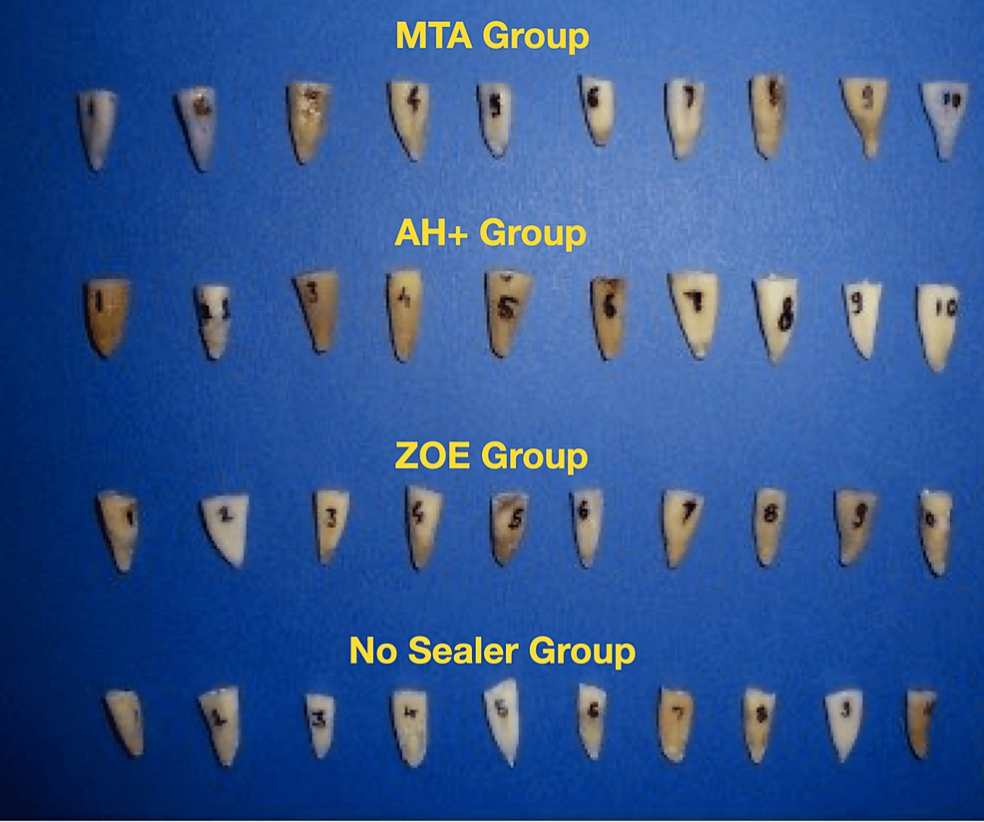
Samples in each group after decoronation MTA, mineral trioxide aggregate; ZOE, zinc oxide eugenol

The teeth were stored in 1% sodium hypochlorite (NaOCl) for 48 hours and in normal saline until further use. Sample preparation involved decorating the teeth at the cemento-enamel junction using a slow-speed diamond disc with constant water cooling (Figure 2).

The resulting root length was standardized to 12 mm using a digital caliper. Canals were accessed using an endo-access bur. The samples were fixed in a wax block, with the root apex extending beyond the wax block, and wrapped in wet gauze to prevent drying and facilitate handling. The working length was determined by placing a No. 15 K-file until it was visible at the apical foramen, and then subtracting 1 mm from this length. Canals with a snug fit of the No. 15 K-file were included in the study. Root canal preparation and obturation were undertaken by a single operator in a standardized method to reduce variations in the final results.

All the canals were prepared in a crown-down fashion using the ProTaper Universal System till file size F3 (tip diameter 0.30 mm). Ethylenediaminetetraacetic acid (EDTA) gel (RC Help) was used as a lubricant with each instrument during canal instrumentation. After each instrument was used, the canals were irrigated with approximately 2 ml of 5% NaOCl. The patency of the apical foramen was verified with a No. 15 K-file after each ProTaper instrument use. Instruments were used for a maximum of five preparations, checked for unwinding, and discarded if necessary. Final irrigation was conducted using 5% NaOCl, 17% EDTA solution, and 3 ml of distilled water. The canals were dried with paper points.

Experimental groups and obturation

The prepared teeth were randomly divided into three experimental groups (n = 10) and one control group (n = 10) as follows: Group A, MTA Fillapex; Group B, AH Plus; Group C, zinc oxide eugenol and Group D, no sealer (control group).

**Figure 3:**
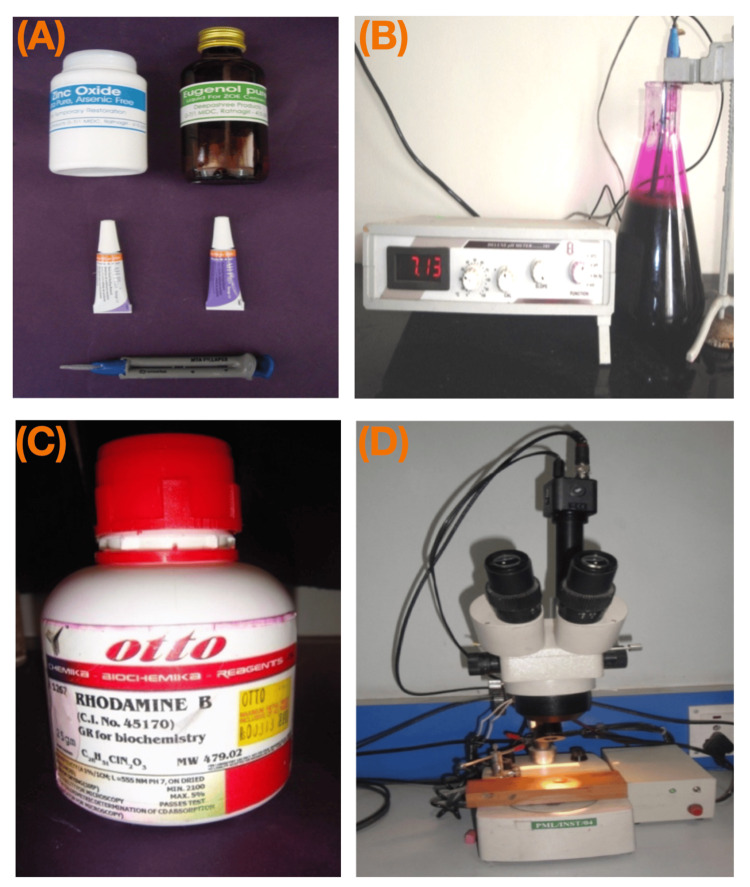
Armamentarium and samples involved in the study: (A) sealers used, (B) rhodamine B dye buffering, (C) rhodamine B, and (D) stereomicroscope

The lateral condensation technique was used for obturation. The endodontic sealers were mixed and used according to the manufacturer's instructions (Figure 3A). For ZOE, one scoop of powder and two drops of liquid were dispensed onto a clean glass slab. The powder was divided into four equal portions and mixed with the liquid incrementally until a homogeneous mix was achieved. For AH Plus, comparable volume units (1:1) of paste A and paste B were mixed to a homogenous consistency on a glass slab using a metal spatula. MTA Fillapex contents were mixed using self-mixing tips attached to a double syringe, and the mixture was dispensed on a mixing pad. Using an EZ-Fill bidirectional spiral, the sealers were introduced into the canal space until the working length. An ISO size 30 gutta-percha cone with a 4% taper was checked for tug back, coated with sealer in the apical part, and slowly introduced into the root until reaching the working length. Lateral condensation was performed using endodontic finger spreaders of 2% taper (ISO size 15, 20, 25) and corresponding standardized gutta-percha points, starting 1 mm short of the working length. Lateral condensation continued until the spreader no longer penetrated beyond the coronal third of the canal. The excess gutta-percha was removed and condensed with a heated plugger. The coronal 2-3 mm of the gutta-percha was removed, and the coronal end of all canals was sealed with Cavit G.

**Figure 4:**
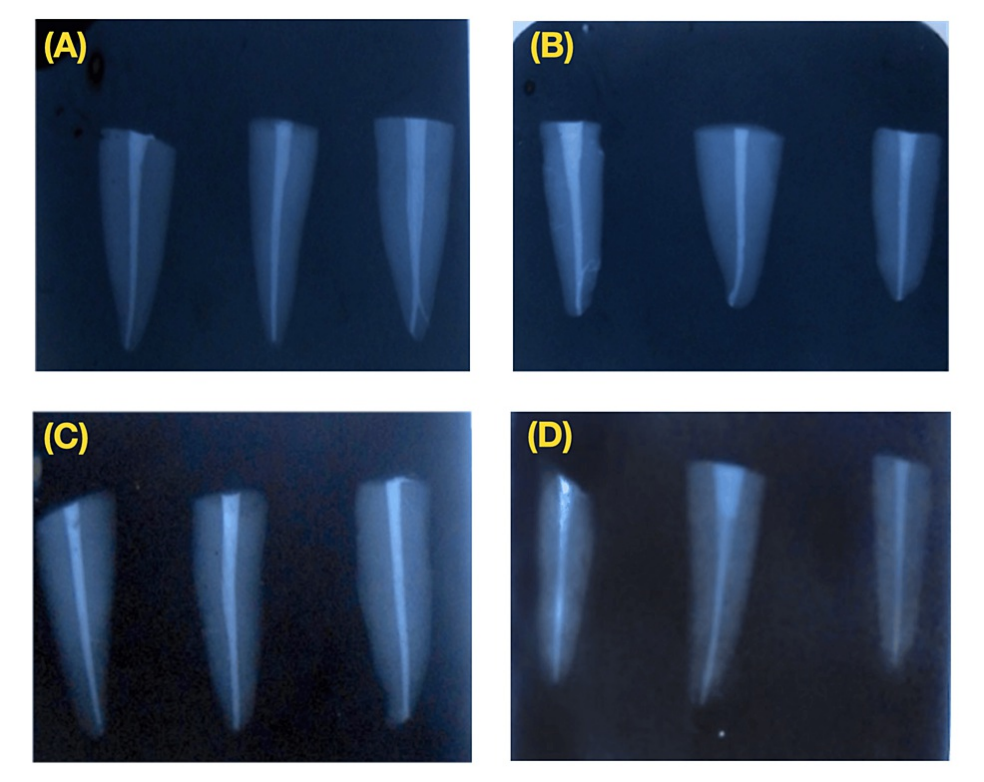
Radiographs of samples from all groups after obturation: (A) MTA Fillapex, (B) AH Plus, (C) ZOE group, and (D) no sealer group MTA, mineral trioxide aggregate; ZOE, zinc oxide eugenol

Radiographs were taken to assess the adequacy of obturation and the presence of any voids (Figure 4). The obturated samples were then placed in normal saline for one week at 37˚C and 100% humidity to ensure the complete setting of the sealers.

Sample processing

**Figure 5:**
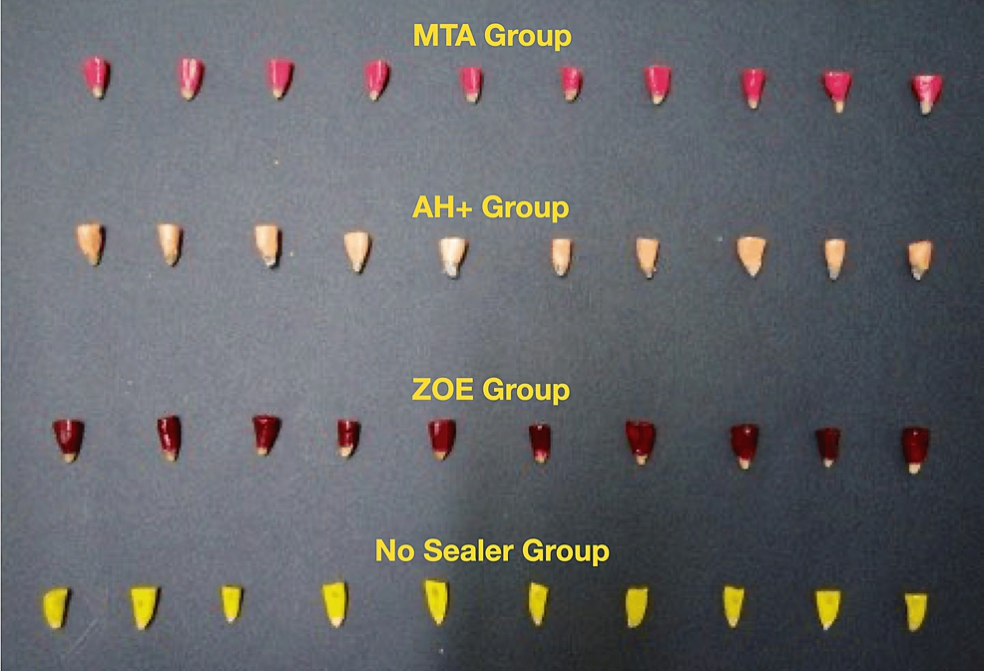
Sample preparation with varnish application in each group MTA, mineral trioxide aggregate; ZOE, zinc oxide eugenol

**Figure 6:**
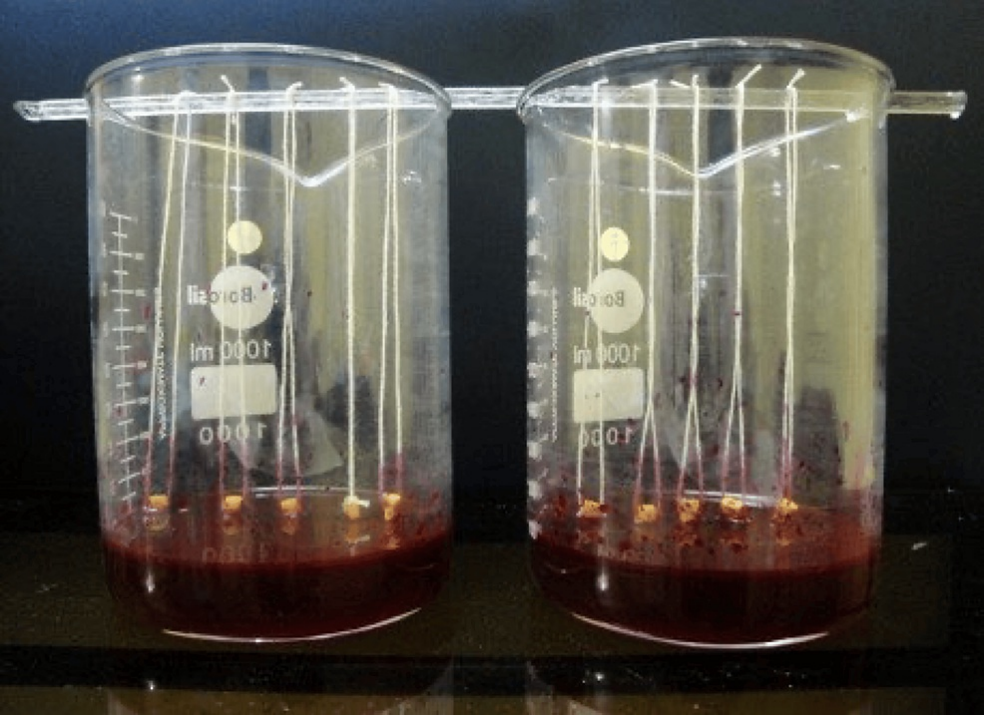
Dye immersion of each sample

After one week, the experimental and control teeth were coated with two layers of nail varnish, exposing the apical 2-mm area around the apical foramen (Figure 5). The teeth were allowed to dry for 48 hours. A 0.2% rhodamine B dye solution (Figure 3B) with a pH of 7.3 was prepared by adding 0.3M phosphate buffer to rhodamine B powder (Figure 3C). The apical portion of the roots was immersed in a 0.2% rhodamine B dye solution with a pH of 7.3 for two days (Figure 6).

**Figure 7:**
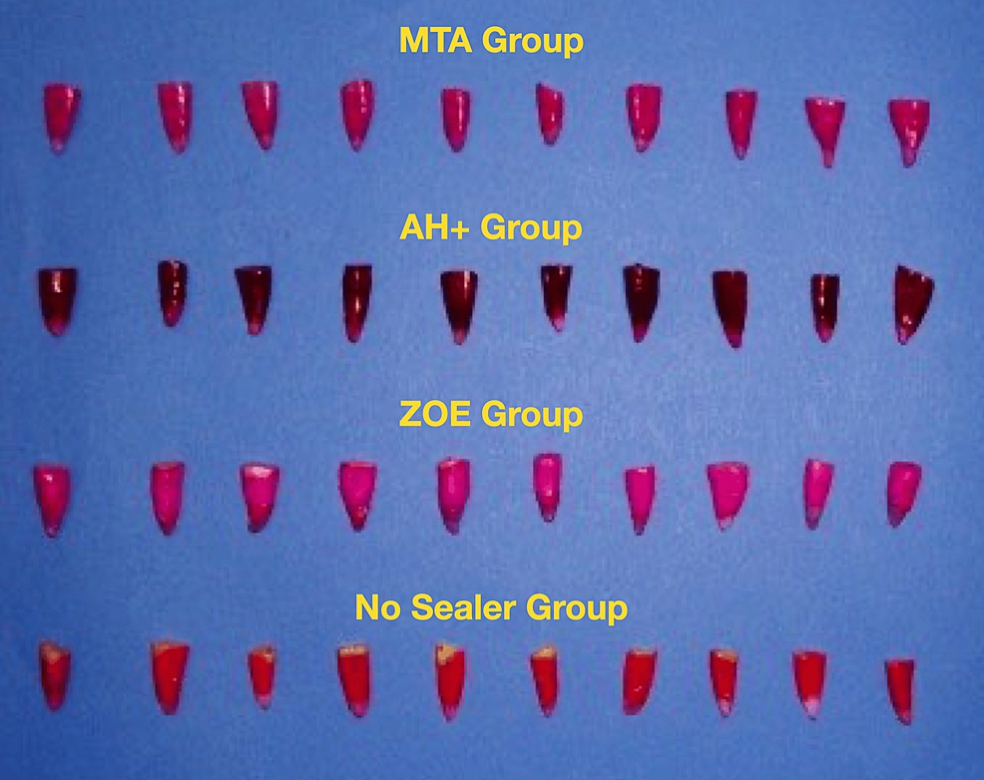
Samples in each group after washing with water for five minutes MTA, mineral trioxide aggregate; ZOE, zinc oxide eugenol

Subsequently, they were washed in running tap water for five minutes and dried at room temperature for 24 hours (Figure 7).

**Figure 8:**
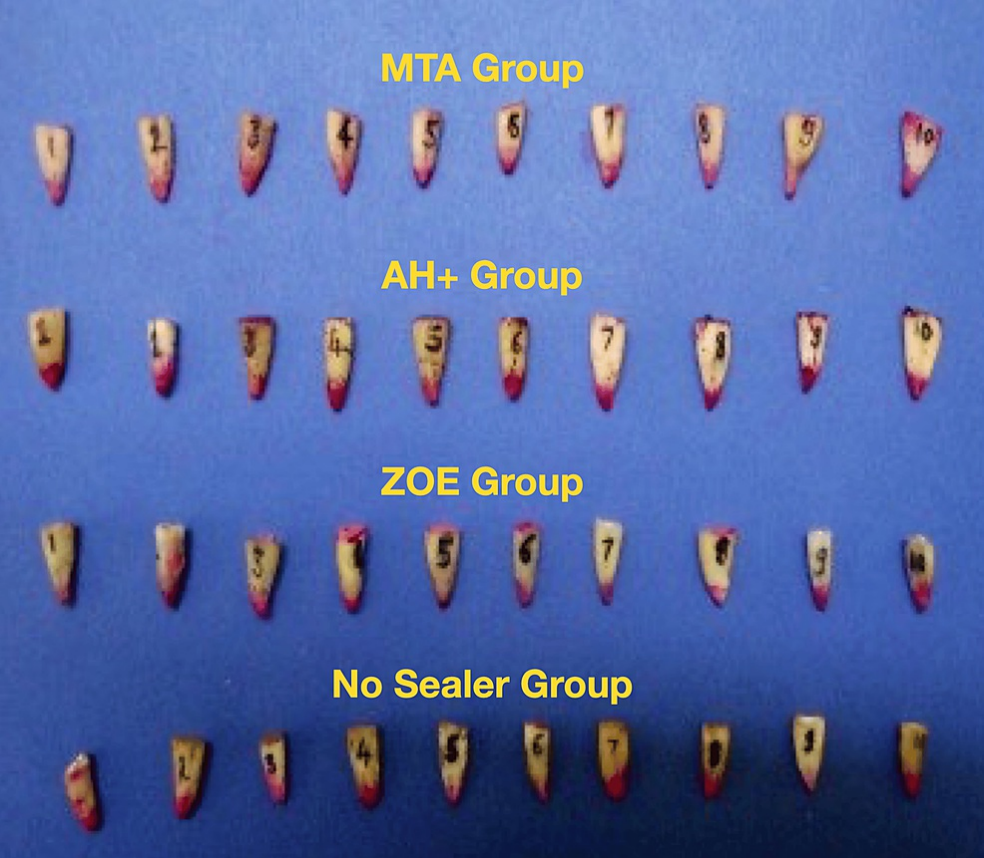
Samples in each group after removal of varnish MTA, mineral trioxide aggregate; ZOE, zinc oxide eugenol

The nail varnish was removed with a scalpel (Figure 8).

**Figure 9:**
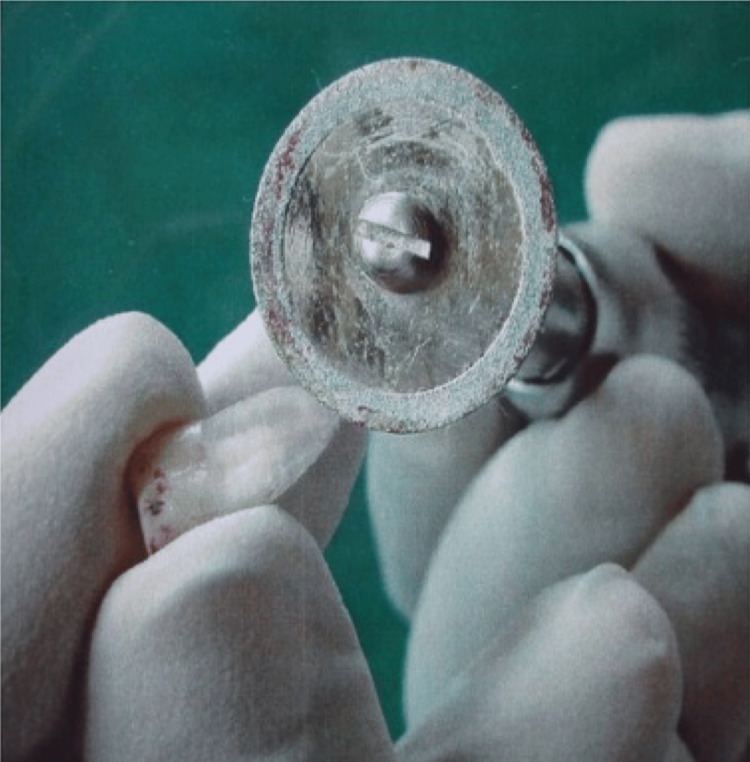
Sample sectioning process using a cylindrical diamond disc

Roots were then ground longitudinally using a cylindrical diamond disc at a high speed with constant water cooling until the root canal filling on one side was reached (Figure 9).

**Figure 10:**
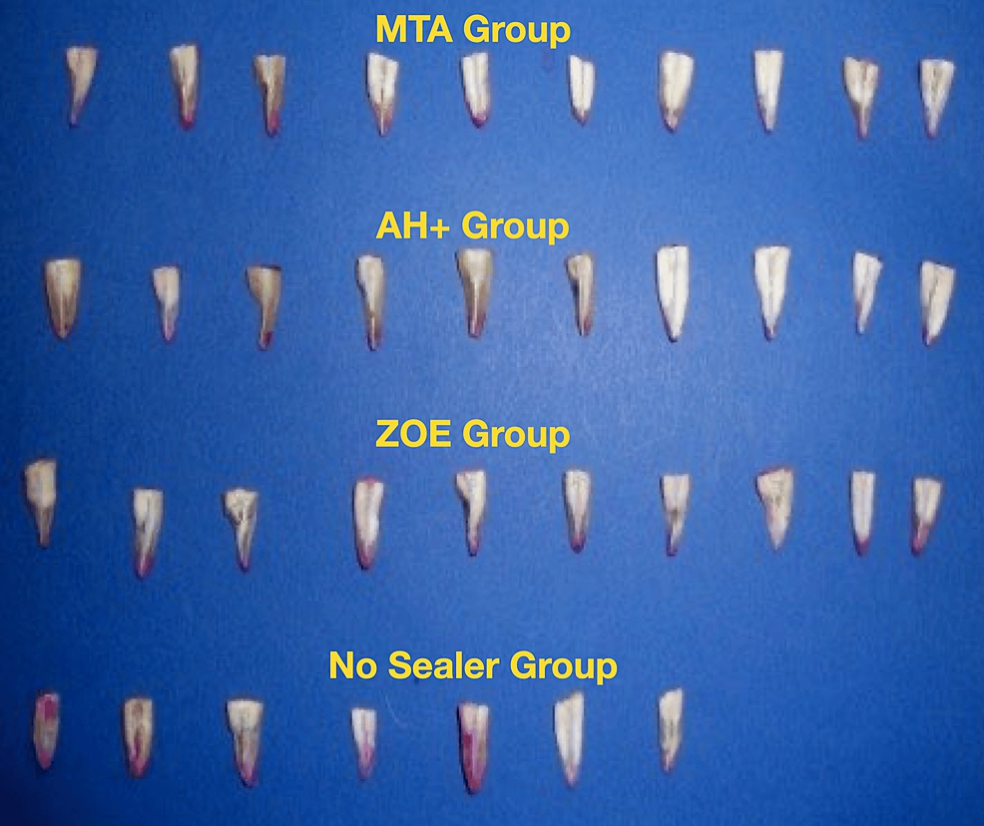
Sectioned samples (total 37) in the four groups (Group A = 10, Group B = 10, Group C = 10, and Group D = 7). MTA, mineral trioxide aggregate; ZOE, zinc oxide eugenol

At the same time, a thin layer of apical dentin was kept intact. The deeper dentin layer was removed using a low-speed diamond disc without water cooling. Three samples from Group D were damaged during this stage and were therefore discarded (Figure 10).

**Figure 11:**
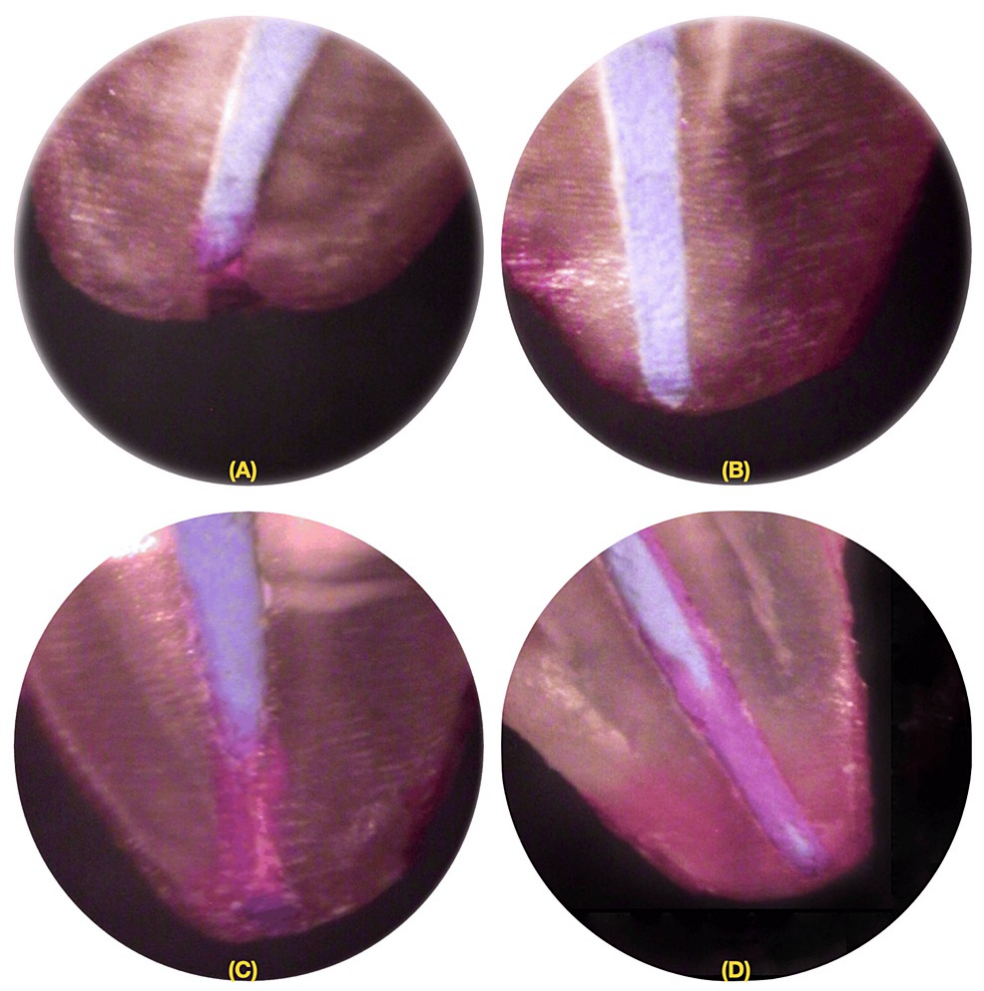
Microscopic view of results from each group: (A) MTA Fillapex, (B) AH Plus, (C) ZOE group, and (D) no sealer group MTA, mineral trioxide aggregate; ZOE, zinc oxide eugenol

The sections were examined under a stereomicroscope (Figure 3D) at 10x magnification, and the depth of dye penetration was measured from the apical end of the gutta-percha to the maximum extent of dye penetration in the coronal direction (Figure 11).

Two observers simultaneously evaluated each sample. The results were tabulated and subjected to parametric one-way analysis of variance (ANOVA) and Student's unpaired t-tests for statistical analysis.

## Results

Table 2 encapsulates the linear penetration of rhodamine B dye (in mm) across dental samples. The data, recorded by two independent observers, provides insights into potential variations among the observed groups (A, B, C, and D). Notably, Group D exhibits fewer observations due to the loss of teeth during sectioning.

**Table 2:** Readings recorded by two observers in each group for the same sample This table showcases the linear penetration of rhodamine B dye (in mm) in dental samples. Two independent observers assessed each sample in Groups A, B, C, and D. Group D had seven observations due to tooth loss during sectioning, as indicated by the "-" symbol.

Sample no.	Group A		Group B		Group C		Group D	
	Observer 1	Observer 2	Observer 1	Observer 2	Observer 1	Observer 2	Observer 1	Observer 2
1	0.3	0.4	0	0	1.8	1.8	11	11
2	0	0	0.4	0.5	3.4	3.4	6.8	6.7
3	0.3	0.3	0	0	2.7	2.7	9.3	9.3
4	0	0	0	0	2	2.1	7.8	7.7
5	0.5	0.4	0	0	4.1	4.2	3.8	3.9
6	0	0	0	0	3	3	2.4	2.5
7	0	0	0	0	2.7	2.7	3.7	3.8
8	0.3	0.3	0.8	0.8	1.5	1.5	-	-
9	0.4	0.4	0	0	1.5	1.6	-	-
10	0	0	0	0	0.7	0.8	-	-

Means of observations (in mm) for apical conditions within each group are presented in Table 3. The nuanced variations are evident, with Group D displaying diverse means. Instances of missing values signify observations not recorded, often attributed to tooth loss during sectioning.

**Table 3:** Means of observations by each group This table presents the means of observations (in mm) for apical conditions within each group (A, B, C, and D). Group A exhibits a range from 0 to 0.45 mm, Group B displays limited observations with Sample 2 measuring 0.45 mm, Group C shows a more diverse range from 0.75 to 4.15 mm, and Group D presents a remarkably diverse set with means ranging from 2.45 to 11 mm. The "-" symbol denotes instances where observations were not recorded due to tooth loss during sectioning.

Sample no.	Means of observations by each sample in each group			
	Group A	Group B	Group C	Group D
1	0.35	0	1.8	11
2	0	0.45	3.85	6.75
3	0.3	0	2.7	9.3
4	0	0	2.05	7.75
5	0.45	0	4.15	3.85
6	0	0	3	2.45
7	0	0	2.7	3.75
8	0.3	0.8	1.5	-
9	0.4	0	1.55	-
10	0	0	0.75	-

Table 4 delves into a comprehensive exploration of apical leakage across Groups A, B, C, and D, encompassing essential statistical measures such as observations, means, sum of squares, variance, and standard deviations. These parameters collectively contribute to a nuanced understanding of the distribution and characteristics of apical leakage within and between the analyzed groups.

**Table 4:** Descriptive statistics of all four independent groups This table provides detailed descriptive statistics for apical leakage in Groups A, B, C, and D. Group A has an average apical leakage of 0.18 mm with a low variance (0.037) and a standard deviation of 0.1947. Group B shows a mean leakage of 0.125 mm with a slightly higher variability (variance = 0.076) and a standard deviation of 0.276. Group C demonstrates a substantial increase in the mean leakage at 2.405 mm, a higher variance of 1.16, and a wider spread denoted by a standard deviation of 1.0771. Group D, with the most increased mean leakage at 6.407 mm, showcases a substantial variance of 10.107 and a significant dispersion of observations (standard deviation ±3.1792).

Groups (k)	Group A	Group B	Group C	Group D	Total
Observations (N)	10	10	10	7	37
Sum (∑X)	1.8	1.25	24.05	44.85	71.95
Mean	0.18	0.125	2.405	6.4071	1.945
Sum of squares (∑X^2^)	0.665	0.8425	68.2825	348.0025	417.7925
Variance	0.037	0.076	1.16	10.107	7.718
Standard deviation (±)	0.1947	0.2761	1.0771	3.1792	2.7783

The results of a one-way ANOVA are detailed in Table 5, offering insights into the variability among group means. The F-statistic and associated p-value highlight the statistical significance of these differences. This table serves as a critical component in affirming the distinctions in the apical leakage across the diverse groups.

**Table 5:** One-way ANOVA for all groups The results of one-way ANOVA show a significant F-statistic of 31.388 (p < 0.01), indicating a substantial variability among group means. Between groups, the sum of squares (SS) is 205.7673, with 3 degrees of freedom (v), resulting in a mean square (MS) of 68.5891. Within groups, the SS is 72.1116, with 33 degrees of freedom, leading to an MS of 2.1852. The total variation is captured in the total SS of 277.8789 with 36 degrees of freedom.

Source of variation	SS	Degree of freedom (v)	MS	F-statistic	p-value
Between groups	205.7673	3	68.5891	31.388	8.78E-10
Error within groups	72.1116	33	2.1852	-	-
Total	277.8789	36	-		

In Table 6, the application of Tukey's honestly significant difference (HSD) test further refines our understanding. The Q-statistic and p-values associated with pairwise comparisons between groups indicate which pairs exhibit statistically significant differences in the apical leakage.

**Table 6:** Tukey's honestly significant difference (HSD) results The results of Tukey's HSD test reveal critical values for Tukey-Kramer HSD Q statistics based on the significance level (α) of 0.01 and 0.05. Pairwise comparisons show significant differences in the apical leakage between certain groups. Group D exhibits the most increased apical leakage, where obturation was performed without any sealer. * and ** denote significance levels.

Groups Pair	Tukey's HSD Q Statistic	Tukey's HSD p-value	Tukey's HSD inference
Group A vs. B	0.11	0.99982	Insignificant
Group A vs. C	4.52	0.01534	p<0.05*
Group A vs. D	12.66	0.00000	p<0.01**
Group B vs. C	4.64	0.01256	p<0.01**
Group B vs. D	12.77	0.00000	p<0.01**
Group C vs. D	8.14	0.00001	p<0.01**

**Figure 12:**
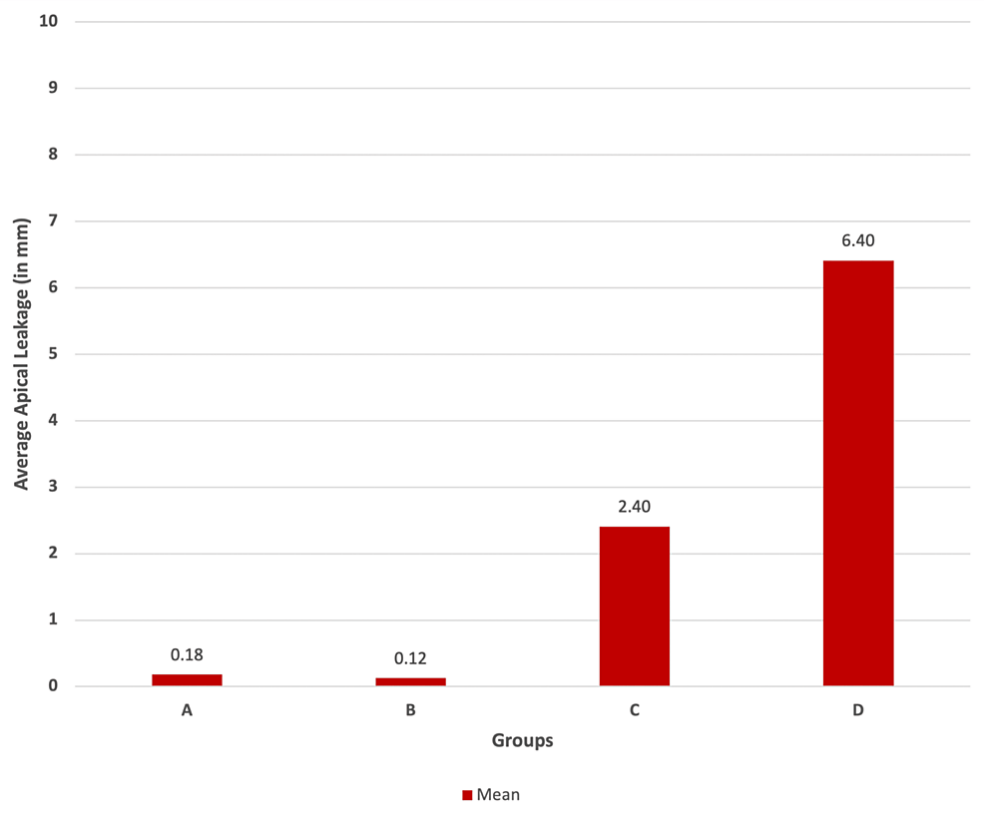
Clustered column chart of the average apical leakage in all groups: Group A, MTA Fillapex; Group B, AH Plus; Group C, zinc oxide eugenol and Group D, no sealer (control group) This clustered column chart visually illustrates the average apical leakage (in mm) with error bars across the four groups, aiding in the visual comparison of means and their associated variability. MTA, mineral trioxide aggregate; ZOE, zinc oxide eugenol

Complementing these tables, Figure 12 visually encapsulates the average apical leakage (in mm) with error bars across the four groups, allowing for a visual comparison of apical characteristics within each group.

## Discussion

Stagnation of fluid within the apical canal results in deteriorated fluid quality, creating toxins that prompt periapical inflammation. The movement of tissue fluids, microorganisms, and toxins via the apical foramen and dentinal walls can lead to endodontic failure [12]. To ensure effective treatment, it is vital to achieve full root canal obturation using an inert filling substance and establish a secure seal at the apex to avert fluid leakage [4]. However, inherent gaps in obturation materials necessitate using endodontic sealers to enhance the seal by filling irregularities and gaps between the filling material and canal walls. The optimal root canal sealer should offer sufficient adhesion, biocompatibility, radio-opacity, stain resistance, dimensional stability, and bactericidal properties or bacteria growth inhibition. It should be easily mixed, introduced, and potentially removed, non-irritating to periapical tissues, and possess a slow setting time to ensure ample working duration. Various sealer types are employed in endodontic practice, each with its own merits and drawbacks [13].

Zinc oxide eugenol sealers, with extended antibacterial effects, result from zinc oxide and eugenol chelation. Moisture is crucial for their setting, as the fully dehydrated zinc oxide paste remains unhardened. They consist of weakly linked zinc oxide grains in a zinc eugenolate matrix. Exposure to water leads to hydrolysis, releasing eugenol and zinc hydroxide. Despite prolonged antibacterial effects, ZOE sealers have moderate adhesion, higher solubility than epoxy amine sealers, and periapical tissue cytotoxicity, driving the development of eugenol-free alternatives [13].

Initially suggested in 1954, AH26, an epoxy amine resin-based sealer, underwent modifications to yield AH Plus, preserving its benefits while overcoming drawbacks such as tooth discoloration and formaldehyde release [14]. AH Plus is a thermoplastic root canal sealer that closely adapts to the canal walls. AH Plus conforms closely to canal walls, ensuring dimensional stability and minimal shrinkage [6]. AH Plus exhibits minimal solubility compared to other sealers and is recognized for its favorable characteristics, which encompass durability, antibacterial attributes, radiopacity, and minimal contraction upon hardening [15].

Fillapex® is a novel root canal sealer that incorporates mineral trioxide aggregate and other components to achieve an appropriate consistency for root canal therapy [8]. Its formulation includes natural resin, salicylate resin, diluting resin, bismuth trioxide, nanoparticulated silica, MTA, and pigments. According to the manufacturer, Fillapex offers a 35-minute working time, excellent flow, a 130-minute setting time, 77% optical density, 0.1% solubility, and easy handling. However, to our knowledge, no research has compared Fillapex's sealing efficacy with commonly used sealers like AH Plus and ZOE. This study evaluated the sealing capability of MTA Fillapex in comparison to AH Plus and ZOE sealers.

Microleakage transfers bacterial fluids, molecules, or ions between the cavity wall and the utilized restoration substance [2]. Assessing dental material sealing in real-life conditions poses challenges, with extended observation periods and participant dropouts. Clinical investigations may vary due to operator skill and assessment criteria. In vitro methods, such as dye penetration, radioactive isotope penetration, electrochemical leakage tests, bacterial infiltration, SEM, and fluid filtration, are developed to evaluate obturation techniques and fillings. Among these, dye penetration is favored for its simplicity, cost-effectiveness, and widespread use in microleakage studies [16].

In this in vitro study, we used the dye penetration method to assess sealer sealing capabilities. Human maxillary incisors with a single, straight, and open canal were employed to standardize anatomy, and their length was adjusted to 12 mm after crown removal. Following established guidelines, the working length was 1 mm short of the apical foramen [17]. Our study also employed the evidence-based crown-down instrumentation approach using the ProTaper system, offering improved flexibility, cutting efficiency, reduced torsional stress, and minimized file use for canal shaping [18].

One operator consistently prepared all canals using a standard protocol to mitigate variability. During preparation, NaOCl and EDTA were used. NaOCl served its antibacterial and tissue-dissolving properties, while EDTA cleared the smear layer and opened dentinal tubules to facilitate better sealer penetration. Paper points were used for drying to enhance the bond between the sealer and root canal dentin [19]. Experimental sealers were applied using the EZ-Fill bidirectional spiral technique, allowing for a simultaneous coronal and apical sealer flow while preventing apical extrusion [20]. Gutta-percha lateral compaction was employed for obturation due to its recognized sealing efficiency. After obturation, specimens were humidified for a week to let sealers set. Nail varnish was used for external root sealing, and dye penetration was expected mainly through apical foramina [21]. Rhodamine B dye was selected for its compatibility with alkaline substances and high dentin permeability. Rhodamine B is suitable for dye penetration studies due to its small particle size, effective diffusion in dentinal tubules, low solubility, non-reactivity with hard tissue, and ease of visualization [22].

After 48 hours of exposing the samples to dye, we longitudinally ground them using a cylindrical diamond disc until reaching the root canal filling on one side. A thin layer of apical dentin was retained to prevent dye removal, while the deeper dentin layer was removed at a low speed without water cooling to minimize dye washout [11]. The sections obtained were examined under a 10x magnification stereomicroscope, and the dye penetration depth was measured from the apical end of the gutta-percha to the maximum extent of dye infiltration towards the coronal direction. Two evaluators assessed each sample, and the dye penetration depth indicated root canal system leakage.

This research compared the sealing abilities of MTA Fillapex, AH Plus, and ZOE sealers. Dye penetration was evident in most samples, and statistical analysis revealed that AH Plus had the least linear dye penetration, followed by MTA Fillapex, ZOE, and the positive control group [23]. Earlier studies have consistently demonstrated better sealing with resin-based sealers like AH Plus than ZOE-based ones [9,24].

This study discovered no statistically significant distinction in the sealing ability between AH Plus and MTA Fillapex. The literature also reports similar sealing effectiveness between AH Plus and MTA [25]. However, MTA Fillapex showed higher leakage than AH Plus after one week, but its sealing performance improved significantly after four weeks, surpassing AH Plus [26]. Over a more extended period, AH Plus continued to demonstrate superior sealing [27].

Fillapex® surpasses the ZOE sealer in sealing performance thanks to its MTA base, which promotes complex tissue deposition through calcium and hydroxyl ion release. The setting expansion of MTA potentially closes gaps and applies pressure to margins, further improving the seal. Additionally, Fillapex's attributes contribute to its superior performance, including low solubility (0.1%), high flow rate, minimal film thickness, and paste-paste formulation akin to AH Plus [28].

Our study demonstrated that sealing prowess of MTA Fillapex is comparable to AH Plus and stronger than the ZOE sealer. This correlates with findings from another study, where MTA Fillapex showcased improved effectiveness and sealing in the cervical and middle sections, similar to our apical third results [29].

The ZOE sealer (Group C) displayed the highest leakage, possibly due to moderate adhesive qualities, rapid setting leading to debonding or fracture [23], and higher solubility than AH Plus. Voids commonly associated with hand-mixed cement may also contribute to the increased leakage of the ZOE sealer.

The control group without any sealer (Group D) demonstrated the highest leakage, consistent with the existing literature [10,24], underscoring the significance of sealers in successful root canal obturation. While some MTA Fillapex and AH Plus samples showed no leakage, others did not entirely seal the apex [30]. The sealing ability can also be influenced by materials and obturation techniques [4]. This in vitro study's limitations preclude evaluating factors like periapical fluid in clinical sealing ability. In conclusion, selecting sealers with superior sealability is pivotal for successful endodontic outcomes.

Limitations

Acknowledging the limitations of this laboratory study is vital. First of all, the results originated from a controlled experimental setup that may not fully replicate the complexities of actual clinical scenarios. Factors such as moisture, dentinal tubules, and interactions with periapical tissues were not considered. Furthermore, evaluating apical leakage using dye penetration provides a restricted understanding of the sealers' sealing efficacy in dynamic clinical situations.

Future directions

Further investigations should focus on developing and creating novel materials exclusively tailored as root canal sealers, aiming for complete elimination of apical leakage. These sealers must undergo rigorous laboratory assessment and live studies to gauge their sealing efficiency and biocompatibility. Future research should also explore how various clinical factors, such as moisture, dentinal tubules, and bacterial challenges, impact the sealing capability of root canal sealers. Additionally, extensive clinical trials are essential to gauge these innovative sealers' real-world performance and success rates in practical endodontic treatments. By addressing these elements, researchers can contribute to advancing endodontic methods and enhancing the sustained results of root canal treatments.

## Conclusions

This in vitro study concluded that irrespective of the sealer used, dye infiltration into the root canal occurred across all groups. AH Plus demonstrated the most minor leakage, emphasizing its superior sealing effectiveness. The apical leakage of both AH Plus and MTA Fillapex displayed no significant disparity, indicating comparable sealing attributes. In contrast, the ZOE sealer exhibited the highest leakage among the tested sealers. These outcomes emphasize the importance of combining root canal sealers with core materials during obturation to mitigate apical leakage. This underscores the crucial role of selecting an appropriate sealer to attain successful outcomes in endodontic procedures.

## References

[1] Schilder H (1967). Filling root canals in three dimensions. Dent Clin North Am.

[2] Muliyar S, Shameem KA, Thankachan RP, Francis PG, Jayapalan CS, Hafiz KA (2014). Microleakage in endodontics. J Int Oral Health.

[3] Gutmann JL (2016). Grossman's Endodontic Practice - 13th Edition. J Conserv Dent.

[4] Li GH, Niu LN, Zhang W (2014). Ability of new obturation materials to improve the seal of the root canal system: a review. Acta Biomater.

[5] Haghgoo R, Ahmadvand M, Nyakan M, Jafari M (2017). Antimicrobial efficacy of mixtures of nanosilver and zinc oxide eugenol against Enterococcus faecalis. J Contemp Dent Pract.

[6] Ørstavik D, Nordahl I, Tibballs JE (2001). Dimensional change following setting of root canal sealer materials. Dent Mater.

[7] Schwartz RS, Mauger M, Clement DJ, Walker WA III (1999). Mineral trioxide aggregate: a new material for endodontics. J Am Dent Assoc.

[8] Vitti RP, Prati C, Silva EJ (2013). Physical properties of MTA Fillapex sealer. J Endod.

[9] Oguntebi BR, Shen C (1992). Effect of different sealers on thermoplasticized gutta-percha root canal obturations. J Endod.

[10] Limkangwalmongkol S, Abbott PV, Sandler AB (1992). Apical dye penetration with four root canal sealers and gutta-percha using longitudinal sectioning. J Endod.

[11] Ahlberg KM, Assavanop P, Tay WM (1995). A comparison of the apical dye penetration patterns shown by methylene blue and India ink in root-filled teeth. Int Endod J.

[12] Wu MK, Wesselink PR (1993). Endodontic leakage studies reconsidered. Part I. Methodology, application and relevance. Int Endod J.

[13] Desai S, Chandler N (2009). Calcium hydroxide-based root canal sealers: a review. J Endod.

[14] Athanassiadis B, George GA, Abbott PV, Wash LJ (2015). A review of the effects of formaldehyde release from endodontic materials. Int Endod J.

[15] Marciano MA, Guimarães BM, Ordinola-Zapata R (2011). Physical properties and interfacial adaptation of three epoxy resin-based sealers. J Endod.

[16] Al-Ghamdi A, Wennberg A (1994). Testing of sealing ability of endodontic filling materials. Endod Dent Traumatol.

[17] Economides N, Liolios E, Kolokuris I, Beltes P (1999). Long-term evaluation of the influence of smear layer removal on the sealing ability of different sealers. J Endod.

[18] Kalra P, Rao A, Suman E, Shenoy R, Suprabha BS (2017). Evaluation of conventional, protaper hand and protaper rotary instrumentation system for apical extrusion of debris, irrigants and bacteria - an in vitro randomized trial. J Clin Exp Dent.

[19] Nagas E, Uyanik MO, Eymirli A, Cehreli ZC, Vallittu PK, Lassila LV, Durmaz V (2012). Dentin moisture conditions affect the adhesion of root canal sealers. J Endod.

[20] Wu MK, van der Sluis LW, Wesselink PR (2006). A 1-year follow-up study on leakage of single-cone fillings with RoekoRSA sealer. Oral Surg Oral Med Oral Pathol Oral Radiol Endod.

[21] Oliver CM, Abbott PV (2001). Correlation between clinical success and apical dye penetration. Int Endod J.

[22] Vogt BF, Xavier CB, Demarco FF, Padilha MS (2006). Dentin penetrability evaluation of three different dyes in root-end cavities filled with mineral trioxide aggregate (MTA). Braz Oral Res.

[23] Wu MK, De Gee AJ, Wesselink PR (1994). Leakage of four root canal sealers at different thickness. Int Endod J.

[24] Limkangwalmongkol S, Burtscher P, Abbott PV, Sandler AB, Bishop BM (1991). A comparative study of the apical leakage of four root canal sealers and laterally condensed gutta-percha. J Endod.

[25] Sönmez IS, Oba AA, Sönmez D, Almaz ME (2012). In vitro evaluation of apical microleakage of a new MTA-based sealer. Eur Arch Paediatr Dent.

[26] Teoh YY, Athanassiadis B, Walsh LJ (2017). Sealing ability of alkaline endodontic cements versus resin cements. Materials (Basel).

[27] Oliveira AC, Tanomaru JM, Faria-Junior N, Tanomaru-Filho M (2011). Bacterial leakage in root canals filled with conventional and MTA-based sealers. Int Endod J.

[28] Hawley M, Webb TD, Goodell GG (2010). Effect of varying water-to-powder ratios on the setting expansion of white and gray mineral trioxide aggregate. J Endod.

[29] Ehsani M, Dehghani A, Abesi F, Khafri S, Ghadiri Dehkordi S (2014). Evaluation of apical micro-leakage of different endodontic sealers in the presence and absence of moisture. J Dent Res Dent Clin Dent Prospects.

[30] Reyhani MF, Ghasemi N, Rahimi S, Milani AS, Barhaghi MH, Azadi A (2015). Apical microleakage of AH Plus and MTA Fillapex® sealers in association with immediate and delayed post space preparation: a bacterial leakage study. Minerva Stomatol.

